# Detecting and Reducing Biases in Cellular-Based Mobility Data Sets

**DOI:** 10.3390/e20100736

**Published:** 2018-09-25

**Authors:** Alicia Rodriguez-Carrion, Carlos Garcia-Rubio, Celeste Campo

**Affiliations:** Department of Telematic Engineering, University Carlos III of Madrid, Avda. Universidad 30, E-28911 Leganés, Madrid, Spain

**Keywords:** human mobility, cell-based location, ping-pong effect, mobility data sets entropy, mobility data sets predictability

## Abstract

Correctly estimating the features characterizing human mobility from mobile phone traces is a key factor to improve the performance of mobile networks, as well as for mobility model design and urban planning. Most related works found their conclusions on location data based on the cells where each user sends or receives calls or messages, data known as Call Detail Records (CDRs). In this work, we test if such data sets provide enough detail on users’ movements so as to accurately estimate some of the most studied mobility features. We perform the analysis using two different data sets, comparing CDRs with respect to an alternative data collection approach. Furthermore, we propose three filtering techniques to reduce the biases detected in the fraction of visits per cell, entropy and entropy rate distributions, and predictability. The analysis highlights the need for contextualizing mobility results with respect to the data used, since the conclusions are biased by the mobile phone traces collection approach.

## 1. Introduction

Human mobility research has regained much interest in recent years, particularly with the advent of smart phones, and there are plenty of works trying to unveil human mobility characteristics from mobile phone traces. Many applications, from mobility models to urban planning [1], traffic forecasting [2], mobility protocols optimization [3], movement prediction [4], or mobile network planning [5], benefit from human mobility analysis, which can be considered as a foundation topic for all these applications. Therefore, it is crucial that the conclusions obtained are not biased by the location history collection technique used to obtain the mobility data.

Many kinds of data sets have been used so far, ranging from Global Positioning System (GPS) [6], cellular [7] or WiFi-based data [8], to the more recent data coming from location-based social networks (LBSNs) [9] or online games [10]. See [11] for a thorough survey of human mobility models and traces.

For extensive traces reflecting global continuous movement in large populations, cellular network data is the preferred choice, since it is difficult to obtain GPS data from many people, WiFi networks are bounded just to certain areas, and the data from LBSNs are highly dependent on the users’ will to publish where they are at every single moment in order to capture a complete movement history. So, the emphasis of this work falls on the cellular network data. The aim of this work is to demonstrate that the way the mobility data are collected noticeably influences the conclusions about mobility, and therefore to invite to reflect on the current results drawn from certain type of mobility data.

Many previous works based on cellular network data use the sequence of locations where the user makes or receives a call or text message (SMS). Cellular wireless operators collect this kind of data, known as Call Detail Records (CDRs), for billing purposes. Therefore, it is possible to obtain an extensive data set of thousands or millions of individuals, which is very useful for significant statistical conclusions. However, these data sets collect user locations only when she makes or receives a voice call, thus hiding locations visited by the user when not. In other words, weights are assigned to locations where she makes calls although those places might not be the most important ones, thereby hiding or biasing user’s mobility features. Therefore, the use of such data sets raises the concern about the reliability of the conclusions drawn from them.

Motivated by these concerns, in this work we analyze two mobility data collection approaches: (i) recording the location of the user using her mobile phone, which knows the identifier of the cell the subscriber is connected to at every moment; and (ii) recording the location of the user only when she makes or receives a phone call or SMS (equivalent to the use of CDRs). This analysis will show the biases suffered by the mobility features due to the location histories drawn from each collection approach. Aiming at reducing them, we propose three filtering techniques to clean the collected data before using it in any application. All the comparisons are backed up by two data sets: a well-known one, collected by Massachusetts Institute of Technology (MIT) students and faculty members during one year; and the University Carlos III of Madrid (UC3M) data set collected during more than one year from different people at different countries who do not share the same space or timetables in their daily life. The two data sets also shed some light in the biases introduced by the intrinsic characteristics of the participants involved in the data collection campaigns.

Specifically, we extend a previous work [12] with the following contributions: (i) in the previous work just the MIT data set was analyzed, here we add a second data set, the UC3M data set, to our analysis; (ii) we transform both data sets (MIT and UC3M) so that we have the same mobility data as if they were collected using the two data collections approaches under study: the one carried out by the mobile phone, and the one carried out by the network; (iii) using the data sets and collection approaches described before, we derived some of the most common mobility features in the literature, highlighting the different statistical distributions obtained in each case; (iv) we propose three filtering techniques to detect and reduce the bias introduced by the collection approaches; and (v) we show how the filtering process affects the observed mobility features under study, by comparing the results to the previous ones.

The paper is organized as follows. Section 2 summarizes the related work. Section 3 describes how mobility data is captured using mobile telephony network, highlighting the mobility data collection approaches under study as well as the effects they introduce into the mobility traces. Section 4 exposes the proposed filtering techniques, whereas Section 5 describes the data sets used for the experiments and the mobility features to be considered for the comparative study. Section 6 digs into the comparative study of the mobility features obtained with the different data sets, collection approaches, and filtering techniques, and discusses some consequences derived from the results. Finally, conclusions are drawn in Section 7.

## 2. State of the Art

The growing interest in human mobility generates a myriad of works analyzing many features extracted from distinct mobility data sets. Focusing on the cellular network-based data, this section presents a review of some of the most significant works on the mobility features characterizing individuals or collective behavior extracted from them.

One of the most studied features is the identification of salient locations. [13] analyzed CDRs of 215 individuals to cluster the most visited locations represented by the cellular network base station transceivers (BTSs). These salient locations are important due to the asymptotic characteristic human behavior, studied by [14] using also CDRs, of the individuals returning systematically to some preferred places, and just sometimes exploring new further areas.

Another of the main features analyzed is the displacement length distribution of the users’ movements. In [15] the authors used the CDRs of 100,000 users to study the power-laws driving the distribution of human displacements.

Ref. [16] went a step further by proposing a new metric for mobility. In their study, they used CDR data coming from 50,000 individuals recorded for three months, and studied different entropy estimators, finally proposing the predictability metric, which sets up an upper bound on the best accuracy a location prediction algorithm could ever achieve. [17] extended this work using a more detailed data set including data from multiple sensors (cellular network, GPS, Bluetooth, WiFi) with higher temporal resolution, comparing how different time scales affect predictability.

In recent years, some studies have been published based on large traces from cellular phone service providers. All of them use CDR traces.

Ref. [18] analyzes anonymized CDRs provided by a major cellular phone service provider in Rwanda, with the activity in the provider’s network during more than three years. They use these traces to develop analytical tools that can identify emergency events in real-time.

Ref. [19] and [20] use CDR traces containing voice call, SMS and Internet activities of nearly 1 million users of a mobile operator in the city of Milan, besides GPS of 178 people collected by Microsoft Research Asia under the GeoLife Project, and WiFi traces extracted from logs of radio services, collected through Access Points (APs) in Dartmouth university campus, to analyze city’s Points of Interest (PoI) and study how the individuals move across them. One of the conclusions is that they obtain a comparable average number of PoIs per user, and the same median of the number of places visited per day, from both the GPS and the CDR data sets.

Ref. [7] analyzes a very large CDR trace that contains 8 million users and 5 billion of call events over 12 months in Mexico. Using these traces, they show properties of user movements between calls.

Ref. [21] uses CDR traces containing mobile phone calls collected in different major cities in Brazil and Mexico. They combine these CDR traces with information from social networks (georeferenced tweets) to predict human mobility.

Ref. [22] uses traces of user trajectories extracted from CDR records released by Orange within their D4D Challenges, and by the University of Minnesota. They use these data to propose solutions to the problem of privacy-preserving publishing of spatiotemporal trajectories of mobile subscribers. To this end, they propose an algorithm that generalizes the data in the trace to protect against attacks on trajectories.

As we said in the introduction, CDR data sets collect user locations only when she actively makes or receives calls, thus hiding or biasing user’s mobility features. Some problems of CDR traces have been pointed in the literature.

Focusing on the possible biases in the features studied due to the use of CDRs, [23,24] raise their concern on the potential bias introduced by this data collection approach. In their reasoning, they claim that the users usually chosen to conduct these studies are those with high voice-call frequency, which might not be representative of the real situation. They conclude that this kind of data sets can infer home and work locations, but provide poor results for overall spatiotemporal properties, such as the set of significant locations (those concentrating 90% of visits) and the entropy and radius of gyration. However, they do not provide a clear comparison based on data coming from the different collection approaches to demonstrate their claim. We try to bridge this gap in this paper.

To avoid the problem that only a few number of geographic points are present per user each day due to the time sparsity of the calls, [25] assume a routine behavior, to create from an original 4-month CDR trace, a new 1-week data set, by aggregating each day of the week the geographic positions observed the same week day in the hole original data set. However, this approach is not effective if user behavior is not very rigid.

Another specific problem of cellular or WiFi-based location data sets is what is known as ping-pong effect, which is studied in [26] and will be described in further sections. The authors detected this effect in WiFi networks, and considered two ping-pong scenarios, between two and three coverage areas. When the ping-pong was detected, the coverage areas implicated were clustered into a new one, and then they compared the number of transitions before and after. In [27] another offline method to filter this ping-pong effect is proposed. In our work, these studies will be extended by considering different detection and filtering approaches that could be applied in an online manner in the case of cellular network-based data.

## 3. Cellular-Based Mobility Data Collection Approaches

As stated in the introduction, among the available location data sources, the cellular telephony networks will be the focus of this work. The cellular network choice allows to: (i) have global coverage, so that users can be continuously tracked any place, any time; (ii) have the lowest device power consumption during the collection process (as opposed to WiFi or GPS options), so that the data recording is unnoticeable for users, and thus making it possible to collect data continuously for long periods of time; and (iii) avoid relying on the user to reveal her location constantly for the data collection (as opposed to LBSNs), so that the data collection is a transparent process for the user, thus avoiding any bias introduced by the users’ willingness to actively record their mobility data.

In the cellular network scenario, the whole space is split into different areas corresponding to the coverage area of each BTS providing the cellular telephony service. Each of these areas, also known as cells, has their own identifier, called cell identifier. For network purposes, the cells are grouped into what is known as location areas, each of them labeled also with different location area code (LAC). Thus, its LAC and cell identifier uniquely identifies each cell. For simplicity, the unique pair (LAC, cell identifier) that represents each cell will be referred to as cellId. Considering this scenario, we focus on the symbolic domain to describe the location of a user, but the translation from this domain to the physical one (based on longitude and latitude) is straightforward if the correspondence between cellIds and BTSs location is known.

**Figure 1:**
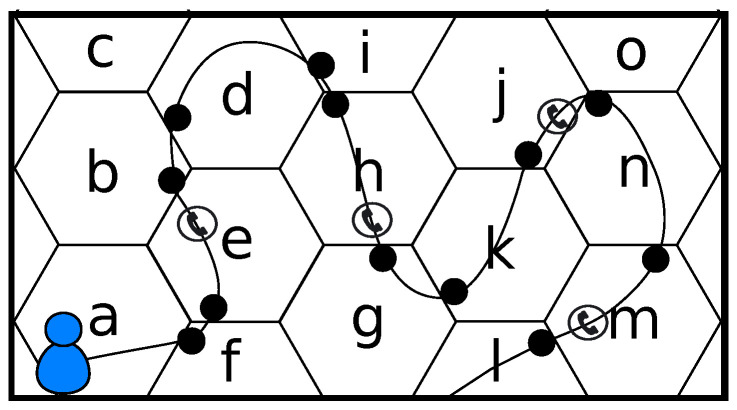
**Data collection approaches**. Mobility data collection approaches in the cellular telephony network.

Both the cellular network and the user’s mobile device are aware of the cell the device is attached to, but in different ways. Therefore, different data collection schemes are possible, resulting in two different types of movement history, location history, or trace, *l*:

- Every mobile device knows the cellId of the cell it is attached to at every moment. Therefore, in this case the location history collects every cell change experienced by the device. This approach will be referred to as **baseline data collection scheme**, $l^{baseline}$. The cases when the mobile device cannot connect to the network (e.g., no coverage in a specific place, network problems…) are not part of the trace, since these cases do not represent any place, just a particular state of the network itself. Only one symbol per location is recorded, thus being not possible to record two consecutive equal cellIds. In the case represented in Figure 1, the location history of the user would be $l^{baseline}=afebdihgkjnml$, i.e., every cell change detected by the device (represented by spots).
- The mobile telephony network collects information regarding the cell the user’s device is attached to every time the user sends or receives voice calls and text messages. This information is stored in what is known as CDR. Therefore, the **CDR-based data collection scheme**, $l^{CDR}$ records a different version of the mobility trace of the user, where only the cellIds of the cells the device was attached to when the user performed some network event (calls, messages) are recorded. In this case, there could be two (or more) subsequent cellIds that are equal, since the user could make a call today and the next one tomorrow at the very same place. With this approach, the user in Figure 1 would have a location history like $l^{CDR}=ehjm$, which corresponds to the sequence of cells where a voice call or a message event took place.

As can be expected, the different data collection approaches will affect the mobility features reflected in the obtained data, potentially biasing the conclusions about human mobility that can be derived from them. These potential biases will be the ones demonstrated in Section 5 and Section 6.

Besides the impact of the collection processes, since we are using a cellular network to locate individuals, we have to take into account how the network operates and how it can influence the data collection process. Each location corresponds to the identifier of the BTS (cell) from which the device receives the best signal strength. However, the subscriber is subject to cell changes due to variations in the radio propagation conditions, or to network operation (e.g., load balancing reasons), even when there is no location change, so that she can be bouncing alternatively between contiguous cells, even without moving. It is important to note that this effect, known as ping-pong effect, takes places only in the baseline collection approach and impacts several mobility features, as it virtually increases the visits to some very specific locations, and adds many fixed patterns throughout the location history that are not tied to the user’s real movement. For this reason, it is important to filter out this effect, and thus we propose some techniques to do so in the next section.

## 4. Mobility Data Filtering Techniques

As described in the previous section, not only the choice of the data collection technique has an impact on the reflected mobility features, but also the working principles of the network may potentially bias the mobility analysis. Section 3 exposed an intrinsic problem of cellular technologies such as WiFi or GSM networks known as ping-pong effect. In order to filter the negative effects of this phenomenon over the mobility data collected, we propose some filtering techniques. All of them consist of a ping-pong sequence detection step, followed by a replacement step.

During the first step, a ping-pong sequence is detected when finding a sequence of events where:

- $l_{n}^{baseline}=l_{n-2}^{baseline}$, until a different symbol from $l_{n}^{baseline}$ and $l_{n-1}^{baseline}$ appears in $l^{baseline}$, for the case of ping-pong between two cells; and
- $l_{n}^{baseline}=l_{n-3}^{baseline}$ and $l_{n-3}^{baseline}\neq l_{n-2}^{baseline}$ and $l_{n-2}^{baseline}\neq l_{n-1}^{baseline}$, and all the consecutive locations corresponding to $l_{n}^{baseline}$, $l_{n-1}^{baseline}$, $l_{n-2}^{baseline}$, until a different locations appears in $l^{baseline}$, for the case of ping-pong among three cells.

We propose three techniques for the replacement step:

- **Representative technique.** Replace the whole ping-pong sequence by the symbols that accounts for a higher number of visits, i.e., the representative_symbol. The problem of this approach is that it does not take into account the adjacency of cells. For example, if the user in Figure 1 has a location history $l^{baseline}=\ldots kjnojonjnojonm\ldots$, and the most visited location is *o*, the filtered sequence would be $l^{representative}=kom$, but there is no real transition between ko or om, as these pairs of cells are not adjacent.
- **Limits technique.** Replace the whole ping-pong sequence by entry_symbol→exit_symbol, where entry_symbol and exit_symbol are the first and last symbols, i.e., the limits of the ping-pong sequence. In the example, the filtered sequence would be $l^{limits}=kjnm$, where every pair of consecutive cells are adjacent. This case solves the problem of the representative technique. However, entry_symbol and exit_symbol will accumulate a large number of visits, which may change the real probability distribution of the visits to each location.
- **Hybrid technique.** Replace the whole ping-pong sequence by entry_symbol→representative_symbol→exit_symbol. If entry_symbol or exit_symbol is equal to representative_symbol, this last one is neglected. In the example above, the filtered sequence would be $l^{hybrid}=kjonm$, which merges the objectives of the previous techniques.

The following section presents the experiments carried out in order to compare the mobility features reflected by the data obtained through the different collection approaches, as well as when filtering it with the techniques described above.

## 5. Experiments Description

This section describes the experiments carried out to check our hypothesis of the direct impact of different mobility data collection approaches on the mobility features under study. The two data sets used are detailed next, together with the mobility features that we are going to focus our study.

### 5.1. Data Sets Description

The experiments were carried out using two data sets. Focusing on the data sets collecting GSM data available for their public use, only the MIT data set [28] was collected using the baseline approach. It also has available the timestamps of calls and SMSs, so that the CDR-based traces can be inferred. The data set is comprised of 95 traces collected in 2004 during 9 months by faculty members and students at the MIT campus.

Although the MIT data set was very useful in analyzing mobility, it was collected several years ago when the cell phone usage was different (e.g., data traffic in mobile phones was rare, whilst phone calls were more frequent). Besides, the participants were all MIT members, who may follow similar patterns since they study or work in the same environment, having similar timetables. There is not enough information to confirm these hypotheses nor to discard them. Therefore, aiming at having a different data set, more recent, and whose participants were not related in locations nor habits, we used another data set that was collected in [29], the UC3M data set.

The UC3M data set is comprised of 25 users, tracked by means of their mobile phones during more than one year. Among the participants, there are people living in five different countries, and working/studying in completely different and independent places, thus not sharing the same space or specific timetable. This data was also collected using the baseline approach, and includes timestamps of calls, so that CDR-based data can also be inferred. The general data about both the MIT and the UC3M data sets is compared in Table 1.

### 5.2. Mobility Parameters to Evaluate

Next, we describe some of the most basic mobility features, which will be further used to illustrate the impact of different data collection approaches on them.

**Amount of movement.** Since we lack of real coordinates to measure the distance covered, we translate this indicator into the number of events (cell changes) made per day.

**Variety of visited locations.** This indicator accounts for the number of different locations visited by the user per day, translated into the number of different cells visited per day.

**Visits distribution.** We measure the fraction of visits concentrated by each location as the percentage of total visits accumulated by each cell, or for certain percentage of different cells, which can unveil the set of important locations. Another point of view is the probability of visiting each location, so we can know how many times we can find a user in each place.

**Randomness.** This parameter is translated into the entropy of a user’s location history. Entropy is an information theory concept that measures the uncertainty about the next event in a sequence (the location history in our case) coming from a realization of a stochastic process. The more random is the sequence, the higher the uncertainty about the next event, and thus the higher entropy value.

We focus on two versions: not taking into account temporal dependencies among locations, for which we use Shannon’s entropy [30] and considering both temporal and spatial correlations, using an estimator of the entropy rate based on [31]. Both entropies are normalized by the maximum possible entropy, given by the logarithm of the maximum number of different locations in the sequence, also known as Hartley entropy, in parallelism with the same metrics used in [16].

**Predictability.** Finally, we use this indicator, defined by [16], bounding the maximum percentage of correct predictions the best algorithm could ever attain, given the specific location history of the user.

## 6. Results and Analysis

In this section, the results of the experiments described in Section 5 are compared. Each mobility feature described in Section 5.2 is calculated for each data set (MIT and UC3M), and compared for the different collection and filtering techniques described in Section 3 and Section 4.

### 6.1. Amount of Movement

**Figure 2:**
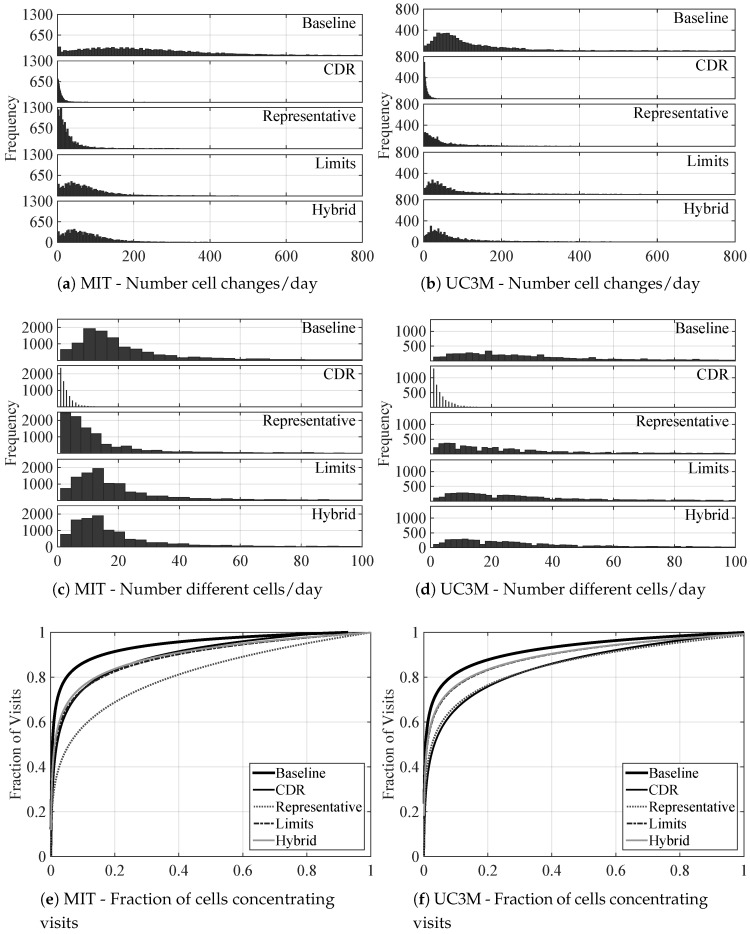
**Comparison of the distributions**. Comparison of (**a**,**b**) the number of cell changes/day for the MIT and UC3M data sets respectively; (**c**,**d**) the number of different cells visited/day for the MIT and UC3M data sets respectively; and (**e**,**f**) the fraction of visits concentrated in each percentage of cells, averaged through the population for the MIT and UC3M data sets respectively, all of them for each data collection approach and filtering technique.

In order to have a general idea of the distribution of this metric, each movement trace is split into days and the number of cell changes performed during each day is accounted. Figure 2 shows the distributions drawn from the aggregation of all days for all users, for the MIT data set in Figure 2a, and for the UC3M data set in Figure 2b. For each subfigure, five plots are presented, displaying the results of the metric when calculated over the movement histories resulting from collecting the mobility data using the baseline approach, $l^{baseline}$, the CDR-based approach, $l^{CDR}$, and the three filtering techniques, $l^{representative}$, $l^{limits}$, and $l^{hybrid}$. Focusing on the differences among data collection approaches, $l^{baseline}$ shows a much wider distribution, ranging from one to around 2000 cell changes per day, whilst $l^{CDR}$ leads to more than one order of magnitude smaller maximum values. This difference roots in the fact that $l^{CDR}$ is actually reflecting the number of calls per day, which is usually smaller compared to the cell change rate due to movement of the user, reflected by $l^{baseline}$. Taking a closer look at the median of cell changes per day reflected by $l^{CDR}$, it adds up to 6 for the MIT case, whereas the median is 4 for the UC3M case, which seems a reasonable number of calls a person can make during one day, but provides limited potential to reflect real mobility features of users. Comparing the results for MIT and UC3M users, the former ones seem to move more than UC3M participants do. Focusing on the filtering techniques, the figure shows that the representative technique is the one deleting more mobility-unrelated events, since the final distribution of cellIds recorded per day is the narrowest and centered on the lowest values. The results for the limits and hybrid techniques are very similar, also reducing the values around which the resulting distribution is centered, although not as much as the representative technique. This behavior holds for both data sets. Looking at the actual statistical values of the distributions, at first it seemed that MIT users move more (median of 203 cell changes per day) than the UC3M users (median of 128 cell changes per day). However, after filtering the traces applying the representative technique, the resulting median values are just the opposite: UC3M users move almost double (median value of 29 cell changes per day) than MIT users (median value of 18). However, since the limits and hybrid techniques add more symbols per ping-pong sequence detected, and the MIT data set has such a high amount of these sequences, the median values greatly decrease (from 203 to 61 for the limits technique, and to 66 for the hybrid technique), but they are still greater than the values for the UC3M data set (29 for the limits technique, and 50 for the hybrid technique). This fact highlights the impact of the high number of ping-pong events in the movement histories.

### 6.2. Variety of Visited Locations

Figure 2 shows the distribution of the number of different cells visited per day for the MIT (Figure 2c) and UC3M (Figure 2d) cases and each of the data collection and filtering choices. Starting again by comparing the results obtained for each data collection approach, the same differences than in the previous case can be identified, even more clearly. The distribution of values reflected by $l^{CDR}$ is notably narrower than that drawn from the baseline approach. People usually make calls in the same places, which according to the median value for both data sets are just two different places per day. Again, these data raise the concern on the quality of the mobility data gathered by the CDR-based approach. Regarding the comparison between data sets, whereas the cell changes per day shown a higher median value for the MIT than for the UC3M users, when considering the number of different cells visited per day the scenario is the opposite one: UC3M users visit a median of 29 different cells per day, compared to the 18 of the MIT users. Considering that the MIT users are all students and stuff of the same university, whilst the UC3M users have different jobs and even reside in different countries, it can be normal that MIT users visit less different locations since all of them share the same closed environment. Regarding the number of different cells visited per day before and after filtering, the differences are not very significant. It can be observed a slight displacement of the distributions to lower values, whilst the shape and width remain practically unchanged. Comparing both data sets, the same properties observed before are present now: UC3M users have a wider distribution, before and after filtering, than MIT users, which now seems to be coherent with the number of cell changes after filtering. Looking at the actual statistical values, it can be checked that, indeed, the median values for UC3M users for all filtering techniques are higher (almost double) than for MIT users. With respect to the differences before and after the filtering phase, the reduction of the statistical values is much smoother than in the case of the cell changes per day. For the MIT users, the median drops to half of the value in the baseline case when using the representative technique, whereas for the rest of cases, it just decreases to four cells less. In the case of UC3M data, the reduction is of eight different cells less in the representative technique, and for the rest to four different cells less. These results seem to indicate that the filtering phase respects the diversity of movements, and just filters out events that do not provide any mobility-related useful information.

### 6.3. Visits Distribution

The next feature under study is the fraction of the total number of different cells visited by a user that concentrate different fractions of visits. Figure 2 shows the cumulative distribution for the aggregated population of the MIT (Figure 2e) and UC3M (Figure 2f) data sets. In order to calculate these curves, all the visited cells were sorted out from the most to the least visited ones, for each user. Then, the fraction of cumulative visits was calculated for each cell, which represents certain percentage out of the total number of different visited cells by that same user. Finally, all the data from each user were aggregated and the curve-fitting tool of Matlab was used to get the parameters defining the power curves with a 95% confidence. This same process was performed independently for each data collection and filtering approach. The results remind of a Pareto distribution, where 20% of the different visited cells concentrate 80% of the total visits in the CDR-based approach for both data sets, whereas for the baseline approach this 20% of different visited cells concentrate up to the 90% of the total visits. Thus, a few cells, such as the ones corresponding to home or work, are much more visited, whilst the majority of locations just slightly add up to the total number of visits. When focusing on the results after filtering ping-pong sequences out, which add many virtual visits to certain locations, the 80–20 relation is still present. When using the hybrid or limits filters, the result is 20% of the different visited cells concentrating close to 80% of the visits, both for MIT and UC3M data sets. The representative technique, on the other hand, leads to a slightly different result, depending on the data set. Due to the enormous number of ping-pong events in the MIT data set, after filtering with the representative technique, 20% of the different visited cells concentrate around 70% of the visits, and 80% of the visits are attained. Thus, very few cells concentrating a significant percentage of visits is an inherent property of human mobility, that is only intensified by ping-pong, which make the case sharper for the baseline movement history (20% of cells concentrate around 90% of visits for both data sets).

**Figure 3:**
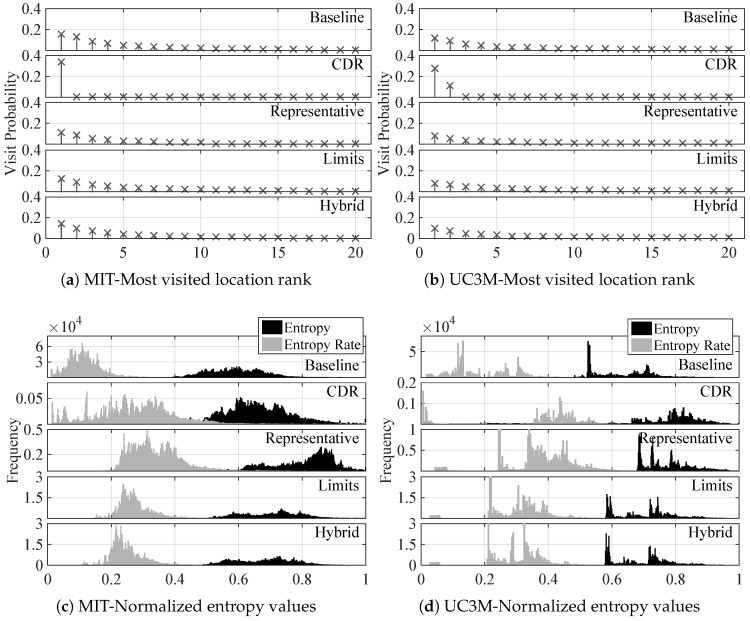

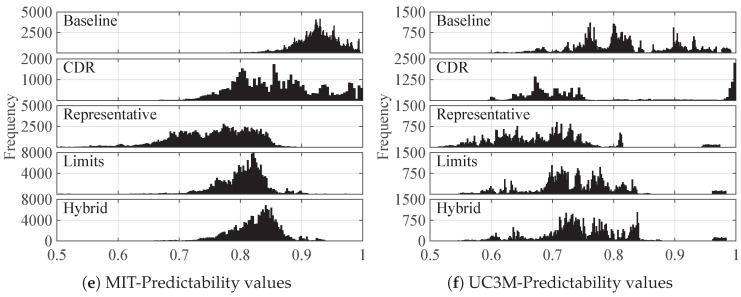
**Comparison of the distributions**. Comparison of (**a**,**b**) the fraction of visits concentrated in each percentage of cells, averaged through the population for the MIT and UC3M data sets respectively; (**c**,**d**) the entropy and entropy rate distributions for the MIT and UC3M data sets respectively; and (**e**,**f**) the predictability distribution for the MIT and UC3M data sets respectively, all of them for each data collection approach and filtering technique.

Another way to visualize this characteristic is by inspecting the visit probability of the most visited cells, which is shown in Figure 3a,b for the MIT and UC3M data sets, respectively. The baseline approach shows a fast decrement of the probability of visit, which drops to half already in the fourth most visited cell in both data sets. This observation reinforces the concern about possible biased predictions due to this noticeable difference between a small group of cells with respect to the rest. The CDR results clearly show that users tend to make calls always from the very same cell, thus the probability of such cell being much higher than the rest, a quite different result than in the baseline case. Regarding the visit probability of the 20 most visited cells represented for the filtered cases, unveil how the difference in the cumulative distributions shown in Figure 2e,f is actually translated into individual cells. As can be seen, the high probability of the most visited cells is clearly diminished for both data sets, which leads to think that a great deal of the ping-pong sequences happened in these most visited cells. Still, the four most visited cells are dominants in terms of visit probability. Another interesting observation, regarding the comparison among the different filtering techniques is that both the limits and hybrid ones lead to a lower decrement in the probability of the four most visited cells. Recall that these two techniques respected the basic structure of the ping-pong sequence by keeping the cells at the limits of the sequence, which correspond to added cells recorded in the movement history with respect to the representative case. These added cells affect mainly the visit probability of the most visited cells, thus reinforcing the hypothesis of the ping-pong sequences mainly being among these most visited cells.

### 6.4. Randomness

Figure 3 shows the aggregated entropy and entropy rate values calculated for both the MIT (Figure 3c) and UC3M (Figure 3d) data sets, and for all data collection and filtering approaches. Regarding the distribution of entropy, the baseline approach leads to a distribution located at lower values than in the CDR case. This means that, without taking into account temporal dependencies, the uncertainty enclosed by $l^{baseline}$ about the next movement of the user is lower than in the CDR case. However, what is significant is the decrease of this uncertainty as soon as temporal correlations (i.e., movement patterns) are considered, that is to say when entropy rate is used to measure the uncertainty. For all the cases in both MIT and UC3M data, the distribution of entropy rate values is located at lower values than the distribution of entropy. If locations are considered as independent events, the uncertainty about what the next location will be is much higher than when locations are considered as a sequence of interrelated events. These results are in consonance with the ones presented in [16]. However, in such work the data considered came from CDRs of an extensive population, whereas our results show noticeable differences between the baseline and CDR collection schemes. For MIT users, the entropy rate distribution is concentrated in the range between 0 and 0.2, whereas for the CDR case is more spread, spanning from 0 to 0.5 values with an irregular distribution. It should be also noted that MIT users were people working or studying in the same university, which can lead to this concentrated distribution in the baseline case due to similar timetables and academic calendars. However, the UC3M users were more diverse and that is reflected into the entropy and entropy rate distributions, which show a much more irregular shape, and in the case of the entropy rate, a wider distribution than in the MIT case. The subplot representing the results drawn from the CDR-based movement histories of the UC3M data set has a very specific distribution concentrated around 0 (people that always make calls from the very same places, thus having no uncertainty on where the next call will be made), and then, larger values than the baseline case (since CDR-based movement history skips all the data between calls, it is more difficult to record the mobility patterns responsible for the low values of entropy rate in the baseline case).

Regarding the results for the filtered traces, the most significant observation is a clear increase of the uncertainty reflected in the shift of both entropy and entropy rate distributions to higher values. This effect is due to the certainty introduced by ping-pong sequences, which are easy to identify and in which it is easy to predict the next cellId. Thus, entropy values decrease, even more taking into account the high percentage of ping-pong events in the sequences. Another observation about the MIT data specifically is how the entropy and entropy rate distributions flatten, leading to more uniform distributions than the resulting one of the baseline case. That corresponds to users behaving differently among them. Ping-pong sequences might also mislead this observation, since these sequences have the very same repetitive behavior for all users and represent a great percentage of the whole movement history. The uniformity obtained by the representative filtering technique disappears when using the limits or hybrid filters. Recall that these two techniques respect the structure of the ping-pong sequence, reducing its length to the minimum. However, the basic structure is always the same, which leads to small versions of ping-pong sequences, and thus slightly lower values of entropy and less uniform entropy rate distributions. The results drawn from the UC3M data are different, since the original distributions are more spread and irregular due to the different users participating in this data set. With these results, it becomes critical to look for useful correct predictions about real movements, since the effect of ping-pong sequences can easily lead to deception.

### 6.5. Predictability

Figure 3 shows the distribution of the values of such metric for the MIT (Figure 3e) and UC3M (Figure 3f) data sets. In this case, the predictability at every single step of each movement history could not be calculated due to the high computational cost, and thus subsequent time needed to process the whole set of available data. For this reason, 2000 samples were randomly chosen over each entire movement history of each user. Starting by comparing the different data collection approaches, in the MIT data set there is a clearer difference among them. The distribution in the baseline case is narrower centered around 93% (as in [16] for the CDR data). However, for CDR-based data, the distribution is wider and concentrated in lower predictability values. The results for the UC3M data set, however, are more irregular (like the entropy rate distribution). For the baseline case, there is not a single value clearly concentrating the majority of values. Since the users follow more different patterns, the predictability of the population varies greatly. In the CDR case, the two sides distribution noticed when analyzing the entropy rate are clearly reflected in the predictability too: one side of the distribution concentrated around high values (people making calls from the same places), and the other side of the distribution located at lower values than the baseline case, due to the lack of information about mobility patterns. For the case of the filtered versions of the baseline traces, the distribution of predictability shifts to lower values, meaning a decrease of the maximum fraction of correct predictions that can be estimated. As explained before, the misleading high predictability of the baseline traces was supported by the striking effect of the ping-pong sequences. Once they are filtered out, the predictability does not exceed values of 0.9, contrasting with the 0.93 value where the maximum of the distribution was centered at in the case of MIT traces. Besides, the representative filtering technique provides a flattening effect over the predictability distribution, also widening the shape. Both the limits and hybrid techniques narrow again the distribution, peaking around 0.8 and 0.85, respectively. Regarding the UC3M data set, the filtering process narrow the distribution. The most significant effect in any case is the shift to lower values that will be directly translated into the predictions results. However, it should be noted that this does not mean a poorer performance of the prediction algorithms, it just means that predicting the real movement of the user is more difficult because the real movements are much more random than when external effects, like ping-pong sequences, which does not incorporate any useful mobility data are included in the traces.

### 6.6. Limitations

The main limitation of our work is that none of the two data sets (MIT and UC3M) contained accelerometer data that would allow us to confirm if the user was really moving or not when a rebound between two or more cells (ping-pong effect) is observed. The tests we have done on mobile phones indicate that the mechanisms we propose correctly filter the rebounds. To completely validate our conclusions, a baseline trace with accelerometer logs would be helpful. It would also be convenient for the data set to contain information of a greater number of users and greater diversity of users (country, age, social stratum, rural and urban environments, etc.) However, it is very difficult to have access to this type of data sets.

## 7. Conclusions and Future Work

This paper demonstrates the impact of the mobility data collection process on the mobility conclusions extracted from studying these data. We compared the results derived from analyzing two data sets: the MIT data set and the UC3M data set. Two data collection processes using the cellular network were considered: (i) the baseline technique, which reflects all the movements of the user, although the associated data sets have less participants, and (ii) CDR-based, which reflects the location of the user only if there is a call going on, but whose data sets comprise millions of users. The comparison of the mobility features under study when using the baseline and CDR-based data unveiled differences in the number of cell changes experienced per day, the number of different cells visited per day, the randomness of users and the consequent predictability of the user’s movements. These results encourage a thorough reflection on the generalizations usually made in mobility studies, which should be contextualized with respect to the data used for the study.

It was also shown that the data coming from the baseline collection approach introduces certain bias due to the ping-pong effect. In order to decrease the bias introduced, an online filtering algorithm was proposed based on a detection scheme and three options for the replacement stage: representative, limits, and hybrid techniques. The representative technique shown to provide the most simplified histories with the lowest bias. However, if the application at hand requires to maintain the real locations structure (e.g., cell adjacency), then the limits or hybrid techniques are better choices. The mobility randomness is highly decreased by the ping-pong effect. Thus, the movement of the user is more random than it may seem without filtering because ping-pong sequences have a fixed structure continuously repeated, which adds a virtually deterministic behavior.

We are currently studying how these biases affect mobility prediction results and trying to improve the algorithms based on the new findings about mobility features.

## Figures and Tables

**Table 1:** **Data sets**. General characteristics of the data sets used for the experiments.

Data Set	MIT	UC3M
**Starting Dates**	2004/09	2013/01
**Participants**	95	25
**Aggregated days**	14,487	5716
